# Change Happens

**DOI:** 10.13023/jah.0401.01

**Published:** 2022-02-13

**Authors:** F. Douglas Scutchfield

**Affiliations:** College of Public Health, University of Kentucky

**Keywords:** Appalachia, journal editing, editorial board

## Abstract

The *Journal of Appalachian Health* is going through some changes that are reflected in the masthead and banner. We say farewell to some colleagues and welcome to the new faces.

The *Journal of Appalachian Health* is going through some changes that are reflected in the masthead and banner. First is the change in the managing editor. We say a sad but fond farewell to Charlotte Seidman. Her reaction to yet another retirement is chronicled by her in a separate comment in this issue of the Journal.^1^ Our readers and I have benefitted from Charlotte’s decision to come out of retirement and be the managing editor of this new Journal. Her knowledge and wisdom of both content and process for publication were key to our successful creation of the journal. She has been present since the creation and has set the standard for continued success of the Journal.

We are pleased and excited to have Charlotte’s replacement on board to do a smooth transition in the open managing editor position. Rachel Dixon is joining us in that role. Like with Charlotte, I have worked with Rachel over several years; she has been my student at least part of the time and has demonstrated her chops in editing and authoring books, chapters, and manuscripts. She is both a scholar herself, as well as being an editor of other’s scholarship and has done outstanding jobs in all of her roles. We welcome her contribution to the rigor and readability of the journal.

Two of our editorial board members have left their current institutions for other pastures. A longtime friend and dear colleague, Linda Alexander, has decided to leave her current post as Associate Dean of Public Health at West Virginia University to occupy a new role as Chief Academic Officer at the Association of Schools and Programs of Public Health. I was involved with hiring Linda to her first appointment in public health at the University of Kentucky, so have valued her wisdom and knowledge over the years. We will miss her guidance and enthusiasm from her work at West Virginia University School of Public Health to her work with the journal.

In her place, representing West Virginia University another long-time colleague and former student, Erik Carlton, has taken on the role of representing WVU on our editorial board. Like Linda, I know Erik well, having served him during his doctoral program at the University of Kentucky school of public health. The decision to put Erik in the WVU editorial board seat on the Journal was the decision of WVU, but I am delighted to have a friend and colleague, as well as a former graduate student, take on the role as a member of our editorial board. Erik is a very bright and effective academician as well as a valued colleague and friend.

Erin Bouldin, who served both as a representative of Appalachia State University as a board member but also one of our Associate Editors has left Appalachia State to go to a position at the University of Utah. We wish her a fond farewell as we do the other three members of our editorial boards’ departure. Appalachia State has named Adam Hege as Erin’s replacement on the board. Adam is associate professor there and has an interest in social determinants and the health of long-haul truck drivers. He is enthusiastic about joining the board and we welcome him.

These new additions to the masthead signal a renewed energy in the Journal; it is always productive to have new sets of eyes and perspectives. We welcome this turnover and the new additions to our masthead. They will bring with them new ideas and directions for the journal. We welcome these new folks and are pleased with the smooth transition that has occurred in our little corner of the publishing world. Join me and the other members of the Journal team in welcoming them to this new role. To our departing colleagues Ave Atque Vale, we thank you for your commitment and diligence in efforts to build the Journal and bring it to its level of excellence.

## References

[1] Seidman CS That’s all she wrote J Appalach Health 2022 4 1 5 8 DOI: 10.13023/jah.0401.02. PMC9200455 35769507

